# Is preoperative chronic kidney disease status associated with oncologic outcomes in upper urinary tract urothelial carcinoma? A multicenter propensity score-matched analysis

**DOI:** 10.18632/oncotarget.16239

**Published:** 2017-03-15

**Authors:** Ho Song Yu, Jun Eul Hwang, Ho Seok Chung, Yang Hyun Cho, Myung Soo Kim, Eu Chang Hwang, Kyung Jin Oh, Sun-Ouck Kim, Seung Il Jung, Taek Won Kang, Dong Deuk Kwon, Kwangsung Park, Soo Bang Ryu, Sung-Hoon Jung, Young Hoe Hur, Joon Hwa Noh, Myung Ki Kim, Ill Young Seo, Chul-Sung Kim, Sung Gu Kang, Seok Ho Kang, Jun Cheon

**Affiliations:** ^1^ Department of Urology, Chonnam National University Medical School, Gwangju, Korea; ^2^ Chonnam National University Research Institute of Medical Sciences, Gwangju, Korea; ^3^ Department of Hemato-Oncology, Chonnam National University Medical School, Gwangju, Korea; ^4^ Department of General Surgery, Chonnam National University Medical School, Gwangju, Korea; ^5^ Department of Urology, Kwangju Christian Hospital, Gwangju, Korea; ^6^ Department of Urology, Chonbuk National University Medical School, Jeonju, Korea; ^7^ Department of Urology, Institute of Wonkwang Medical Science, Wonkwang University School of Medicine, Iksan, Korea; ^8^ Department of Urology, Chosun University School of Medicine, Gwangju, Korea; ^9^ Department of Urology, Korea University College of Medicine, Seoul, Korea

**Keywords:** renal insufficiency, chronic, carcinoma, transitional cell, prognosis

## Abstract

**Purpose:**

The aim of this study was to determine the effect of preoperative chronic kidney disease (CKD) on the prognosis of patients with upper urinary tract urothelial carcinoma (UTUC) who had undergone radical nephroureterectomy (RNU).

**Results:**

The median follow-up period was 31.1 months (interquartile range: 16.2-55.7 months). Among the study patients, 224 patients in the non-CKD group were selected *via* propensity score matching. The median recurrence-free, cancer-specific, and overall survival were significantly shorter for patients with preoperative CKD than for non-CKD patients (*p* = 0.001, *p* = 0.001, and *p* = 0.001, respectively). According to multivariable Cox regression analysis, preoperative CKD was related to worse recurrence-free (hazard ratio [HR]: 1.81, 95% confidence interval [CI]: 1.15-2.86, *p* = 0.011), cancer-specific (HR: 2.44, 95% CI: 1.44-4.14, *p* = 0.001), and overall survival (HR: 1.66, 95% CI: 1.15-2.40, *p* = 0.007).

**Methods:**

A total of 566 patients who underwent RNU at 6 institutions from 2004 to 2014 were retrospectively reviewed. Of these patients, 342 had an estimated glomerular filtration rate (eGFR) ≥ 60 ml/min/1.73 m^2^ (non-CKD group) and 224 patients had an eGFR <60 ml/min/1.73 m^2^ (CKD group). To adjust for potential baseline confounders, 224 patients in the non-CKD group were selected by propensity matching. Clinicopathological variables and survival rates were compared between the 2 groups.

**Conclusions:**

Preoperative CKD appears to be an important independent prognostic factor for oncologic outcomes in patients with UTUC.

## INTRODUCTION

Upper tract urothelial carcinoma (UTUC) is a urothelial malignancy involving the renal pelvis or ureter. UTUCs are rare and account for only 5-10% of urothelial carcinomas [1, 2]. However, at the time of diagnosis, about 60% of UTUCs are invasive compared with 15-25% of bladder tumors [3, 4]. The standard treatment of UTUC is surgery with radical nephroureterectomy (RNU) or distal ureterectomy in select patients. Old age, tobacco consumption, tumor location, multifocality, and waiting time for surgery are known preoperative prognostic factors [5]. Tumor stage and grade, lymph node involvement, lymphovascular invasion, surgical margin, and tumor necrosis are considered significant postoperative prognostic factors [5].

There is emerging evidence that chronic kidney disease (CKD) is related to an increased risk of cardiovascular mortality [6], all-cause mortality [7], and non-cardiovascular mortality [8]. In addition, CKD is common among elderly patients and is associated with cancer risk, particularly in the kidneys and urinary tract [9]. Several studies have examined the prevalence of CKD before and after RNU in UTUC patients [10, 11]. These studies revealed that UTUC is associated with a high risk of the doubling of the serum creatinine levels and/or the occurrence of end-stage renal disease necessitating dialysis following unilateral RNU, and these circumstances would lead to deferred administration of adjuvant chemotherapy.

However, to date, few studies have evaluated the prognostic value of preoperative CKD status on oncologic outcome in UTUC patients. From this perspective, it appeared necessary to focus on the effect of preoperative CKD on oncological outcomes in patients with UTUC. Thus, we analyzed demographic, clinical, and pathologic data of 556 patients with UTUC who underwent RNU at 6 institutions during a period of 10 years for recurrences, cancer-specific survival, and overall survival of UTUC in relation to preoperative CKD status.

## RESULTS

### Patient demographics

Table 1 shows a comparison of baseline patient characteristics between the non-CKD and CKD groups. Before propensity matching analysis, the median patient age was 72 years (interquartile range [IQR]: 65-76) and the median follow-up duration was 31.1 months (IQR: 16.2-55.7). Among all cohorts, the incidence of synchronous bladder tumor was 19.6%, and there was no difference between the two groups.

**Table 1:** Baseline characteristics of overall and propensity score-matched cohort of upper urinary tract urothelial carcinoma patients with preoperative non-chronic kidney disease and chronic kidney disease

Variables	Overall cohorts			Propensity score matched cohorts		
	Non CKD (*n* = 342)	CKD (*n* = 224)	*p* value	Non CKD (*n* = 224)	CKD (*n* = 224)	*p*-value
Age (years), median (IQR)	68 (60-74)	72 (66-77)	0.001^*^	71 (64-75)	72 (66-77)	0.051^*^
Gender (n, %)						
Female	87 (25.4)	78 (34.8)	0.016^†^	68 (30.4)	78 (34.8)	0.364^†^
Male	255 (74.6)	146 (65.2)		156 (69.6)	146 (65.2)	
BMI (mean ± SD, kg/m^2^)	23.7 ± 3.1	23.9 ± 3.0	0.356^*^	23.8 ± 3.1	23.9 ± 3.0	0.756^*^
preoperative serum creatinine (mean ± SD, mg/dL)	0.9 ± 0.2	1.6 ± 1.1	0.001^*^	0.9 ± 0.2	1.6 ± 1.1	0.001^*^
Preoperative eGFR (mean ± SD, mL/min/1.73 m^2^)	82.2 ± 18.9	46.9 ± 11.3	0.001^*^	80.8 ± 19.3	46.9 ± 11.3	0.001^*^
DM	72 (21.1)	63 (28.1)	0.056^†^	55 (24.6)	63 (28.1)	0.453^†^
HTN	121 (35.4)	79 (35.3)	0.978^†^	83 (37.1)	79 (35.3)	0.768^†^
ECOG performance status (n, %)						
0	258 (75.4)	141 (62.9)	0.004^†^	151 (67.4)	141 (62.9)	0.329^†^
1	77 (22.5)	69 (30.8)		66 (29.5)	69 (30.8)	
2	7 (2)	13 (5.8)		7 (3.1)	13 (5.8)	
3	0 (0)	1 (0.4)		0 (0)	1 (0.4)	
Operation method (n, %)						
Open	80 (23.4)	62 (27.7)	0.250^†^	53 (23.7)	62 (27.7)	0.387^†^
Laparoscopic	262 (76.6)	162 (72.3)		171 (76.3)	162 (72.3)	
Tumor location (n, %)						
Renal pelvis	172 (50.3)	86 (38.4)	0.015^†^	98 (43.8)	86 (38.4)	0.511^†^
Upper ureter	42 (12.3)	29 (12.9)		31 (13.8)	29 (12.9)	
Mid ureter	38 (11.1)	42 (18.8)		32 (14.3)	42 (18.8)	
Lower ureter	90 (26.3)	67 (29.9)		63 (28.1)	67 (29.9)	
Hydronephrosis (n, %)						
None	131 (38.3)	35 (15.6)	0.001^†^	51 (22.8)	35 (15.6)	0.273^†^
Mild	88 (25.7)	63 (28.1)		55 (24.6)	63 (28.1)	
Moderate	74 (21.6)	72 (32.1)		70 (31.3)	72 (32.1)	
Severe	49 (14.3)	54 (24.1)		48 (21.4)	54 (24.1)	
Synchronous bladder tumor						
No	276 (80.7)	179 (79.9)	0.817^†^	177 (79.0)	179 (79.9)	0.907^†^
Yes	66 (19.3)	45 (20.1)		47 (21.0)	45 (20.1)	
Tumor size (mean ± SD, cm)	3.4 ± 2.4	3.7 ± 2.6	0.162^*^	3.4 ± 2.3	3.7 ± 2.6	0.162^*^
Multifocality (n, %)						
No	313 (91.5)	204 (91.1)	0.853^†^	205 (91.5)	204 (91.1)	0.867^†^
Yes	29 (8.5)	20 (8.9)		19 (8.5)	20 (8.9)	
Pathologic stage (n, %)						
Tis, Ta	62 (18.1)	22 (9.8)	0.099^†^	33 (14.7)	22 (9.8)	0.595^†^
T1	77 (22.5)	51 (22.8)		52 (23.2)	51 (22.8)	
T2	78 (22.8)	56 (25.0)		54 (24.1)	56 (25.0)	
T3	114 (33.3)	86 (38.4)		77 (34.4)	86 (38.4)	
T4	11 (3.2)	9 (4.0)		8 (3.6)	9 (4.0)	
Pathologic grade (n, %)						
Low	127 (37.1)	55 (22.8)	0.001^†^	81 (36.2)	51 (22.8)	0.003^†^
High	215 (62.9)	173 (77.2)		143 (63.8)	173 (77.2)	
Lymphovascular invasion (n, %)						
No	286 (83.6)	161 (71.9)	0.001^†^	188 (83.9)	161 (71.9)	0.003^†^
Yes	56 (16.4)	63 (28.1)		36 (16.1)	63 (28.1)	
Concomitant CIS (n, %)						
No	307 (89.8)	205 (91.5)	0.559^†^	204 (91.1)	205 (91.5)	0.867^†^
Yes	35 (10.2)	19 (8.5)		20 (8.9)	19 (8.5)	
Adjuvant chemotherapy (n, %)						
No	226 (66.1)	135 (60.3)	0.159^†^	148 (66.1)	135 (60.3)	0.203^†^
Yes	116 (33.9)	89 (39.7)		76 (33.9)	89 (39.7)	
Recurrence (n, %)	38 (11.1)	54 (24.1)	0.001^†^	29 (12.9)	54 (24.1)	0.002^†^
Cancer death (n, %)	30 (8.8)	52 (23.2)	0.001^†^	19 (8.5)	52 (23.2)	0.001^†^
Death from any cause (n, %)	69 (20.2)	79 (35.3)	0.001^†^	46 (20.5)	79 (35.3)	0.001^†^

Abbreviations: IQR, interquartile range; BMI, body mass index; eGFR, estimated glomerular filtration rate; DM, diabetes mellitus; ECOG, Eastern Cooperative Oncology Group; CIS, carcinoma in situ

^*^Mann-Whitney U test

^†^Pearson chi-square test

There were statistically significant differences in patient age, sex, ECOG-PS, tumor location, and hydronephrosis. However, after propensity matching, there were no differences in these variables between the non-CKD and CKD groups (C-statistics ranged from 0.65 to 0.74, mean 0.69). Pathologic grade, lymphovascular invasion, recurrence, cancer death, and death from any cause were found to differ significantly between the non-CKD and CKD groups.

### Impact of CKD on oncological outcomes

**Figure 1:**
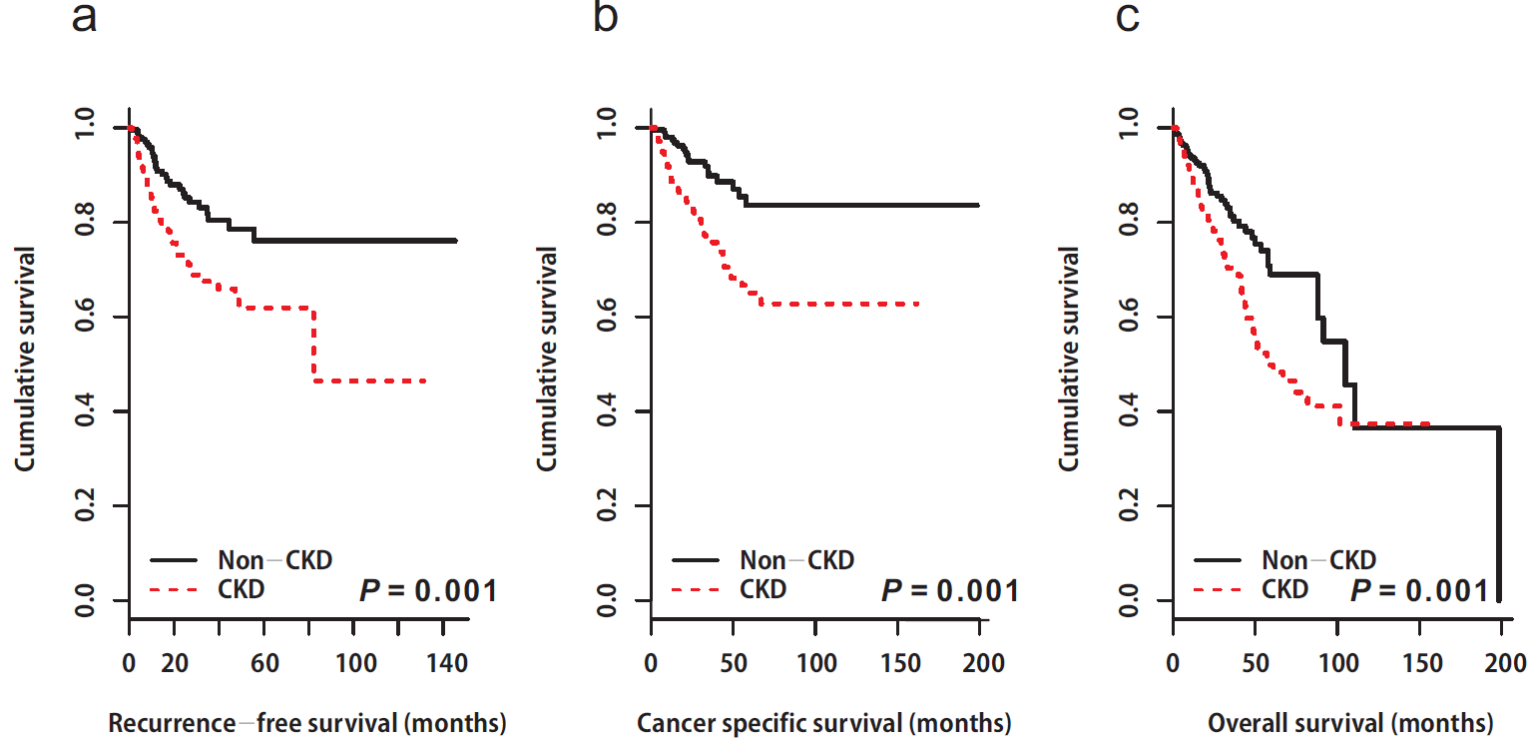
Kaplan-Meier estimates depicting the recurrence-free survival (a), cancer-specific survival (b), and overall survival (c) stratified according to preoperative chronic kidney disease status **a.** Recurrence-free survival. Non CKD; median: not available vs. CKD; median: 82.3 months (95% CI: not available), *p* = 0.001. CKD, chronic kidney disease; CI, confidence interval. **b.** Cancer specific survival. Non CKD; median: not available vs. CKD; median: not available, *p* = 0.001. CKD, chronic kidney disease. **c.** Overall survival. Non CKD; median: 105 months (95% CI: 88.1–not available) vs. CKD; median: 58.0 months (95% CI: 48.9– not available), *p* = 0.001. CKD, chronic kidney disease; CI, confidence interval

A total of 83 (18.5%) patients experienced disease recurrence during the follow-up period. Of these, 19 (4.2%) patients had local recurrence and 64 (14.3%) patients experienced distant metastasis. The 5-year RFS rates after RNU were 76% for the non-CKD group, and 61% for the CKD group. Patients in the CKD group showed poor RFS compared to those in the non-CKD group (non-CKD, not reached *vs*. CKD; median 82.3 months, 95% CI: not available, *p* = 0.001, Figure 1a). Univariable and multivariable Cox regression analyses showed that preoperative CKD was associated with an increased risk of disease recurrence (univariable, HR: 2.12, 95% CI: 1.35-3.34, *p* = 0.001; multivariable, HR: 1.81, 95% CI: 1.15-2.86, *p* = 0.011; Tables 2 & 3).

**Table 2:** Univariable Cox regression analyses predicting recurrence-free, cancer-specific and overall survival in propensity score-matched cohorts with upper urinary tract urothelial carcinoma after radical nephroureterectomy

Variables	Recurrence-free survival		Cancer-specific survival		Overall survival	
	HR (95% CI)	*p* value	HR (95% CI)	*p* value	HR (95% CI)	*p* value
Age (continuous)	0.99 (0.97-1.02)	0.857	1.01 (0.98-1.04)	0.497	1.04 (1.02-1.07)	0.001
Male (vs. Female)	1.48 (0.91-2.44)	0.116	1.38 (0.82-2.34)	0.223	1.23 (0.83-1.82)	0.296
BMI (continuous)	0.92 (0.86-0.98)	0.021	0.94 (0.87-1.01)	0.104	0.91 (0.86-0.97)	0.002
DM	0.96 (0.58-1.59)	0.879	1.61 (0.99-2.59)	0.052	1.19 (0.82-1.74)	0.367
HTN	0.78 (0.49-1.25)	0.313	0.83 (0.51-1.37)	0.486	0.87 (0.59-1.26)	0.464
ECOG-PS 2-3 (vs. 0-1)	1.31 (0.48-3.58)	0.597	1.66 (0.61-4.57)	0.324	2.18 (1.10-4.31)	0.025
Operation method						
Laparoscopic (vs. open)	1.05 (0.64-1.71)	0.857	0.67 (0.41-1.08)	0.100	1.03 (0.71-1.49)	0.876
Tumor location						
Ureter (vs. renal pelvis)	1.02 (0.65-1.59)	0.921	0.84 (0.52-1.34)	0.456	0.75 (0.65-1.35)	0.746
Hydronephrosis (vs. none)	1.11 (0.64-1.94)	0.710	0.96 (0.54-1.70)	0.893	1.05 (0.68-1.63)	0.829
Synchronous bladder tumor	1.09 (0.62-1.87)	0.767	1.30 (0.69-2.03)	0.346	1.03 (0.66-1.60)	0.891
Tumor size (continuous)	1.07 (0.98-1.15)	0.094	1.04 (0.94-1.14)	0.496	1.06 (0.98-1.14)	0.129
Multifocality	1.54 (0.74-3.20)	0.246	1.65 (0.78-3.43)	0.185	1.68 (0.94-2.98)	0.079
Pathologic stage T3-4 (vs. Tis, Ta, T1, T2)	4.35 (2.24-8.42)	0.001	3.67 (1.93-6.98)	0.001	2.56 (1.66-3.95)	0.001
Pathologic grade						
high grade (vs. low grade)	3.70 (1.85-7.39)	0.001	2.96 (1.51-5.78)	0.002	2.03 (1.31-3.14)	0.002
Lymphovascular invasion	4.47 (2.90-6.88)	0.001	3.22 (2.01-5.14)	0.001	2.50 (1.73-3.62)	0.001
Concomitant CIS	1.67 (0.86-3.23)	0.132	1.88 (0.94-3.79)	0.077	1.55 (0.85-2.82)	0.150
Adjuvant chemotherapy	3.30 (2.01-5.19)	0.001	3.51 (2.14-5.74)	0.001	2.54 (1.77-3.63)	0.001
CKD	2.12 (1.35-3.34)	0.001	2.76 (1.63-4.67)	0.001	1.77 (1.23-2.56)	0.002

Abbreviations: HR, hazard ratio; CI, confidence interval; BMI, body mass index; DM, diabetes mellitus; HTN, hypertension; ECOG-PS, Eastern Cooperative Oncology Group Performance Status; CIS, carcinoma in situ; CKD, chronic kidney disease.

**Table 3:** Multivariable Cox regression analyses predicting recurrence-free, cancer-specific, and overall survival in propensity score-matched cohorts with upper urinary tract urothelial carcinoma after radical nephroureterectomy

Variables	Recurrence-free survival		Cancer-specific survival		Overall survival	
	HR (95% CI)	*p* value	HR (95% CI)	*p* value	HR (95% CI)	*p* value
Age (continuous)					1.05 (1.02-1.07)	0.001
BMI (continuous)	0.93 (0.87-1.01)	0.060			0.93 (0.88-0.99)	0.024
DM			1.84 (1.13-2.99)	0.014		
ECOG-PS 2-3 (vs. 0-1)					1.76 (0.85-3.63)	0.128
Tumor size (continuous)					1.06 (0.98-1.14)	0.128
Multifocality					1.53 (0.94-2.51)	0.089
Pathologic stage T3-4 (vs. Tis, Ta, T1,T2)	2.33 (1.14-4.75)	0.020	2.27 (1.11-4.64)	0.024	1.93 (1.07-3.47)	0.029
Pathologic grade						
high grade (vs. low grade)	1.98 (0.96-4.12)	0.064	1.59 (0.76-3.32)	0.211	1.16 (0.73-1.87)	0.530
Lymphovascular invasion	2.93 (1.86-4.64)	0.001	2.14 (1.31-3.49)	0.002	1.82 (1.24-2.68)	0.002
Adjuvant chemotherapy			1.76 (0.88-3.15)	0.157	1.39 (0.91-2.84)	0.132
CKD	1.81 (1.15-2.86)	0.011	2.44 (1.44-4.14)	0.001	1.66 (1.15-2.40)	0.007

Abbreviations: HR, hazard ratio; CI, confidence interval; BMI, body mass index; ECOG-PS, Eastern Cooperative Oncology Group Performance Status; CKD, chronic kidney disease.

**Figure 2:**
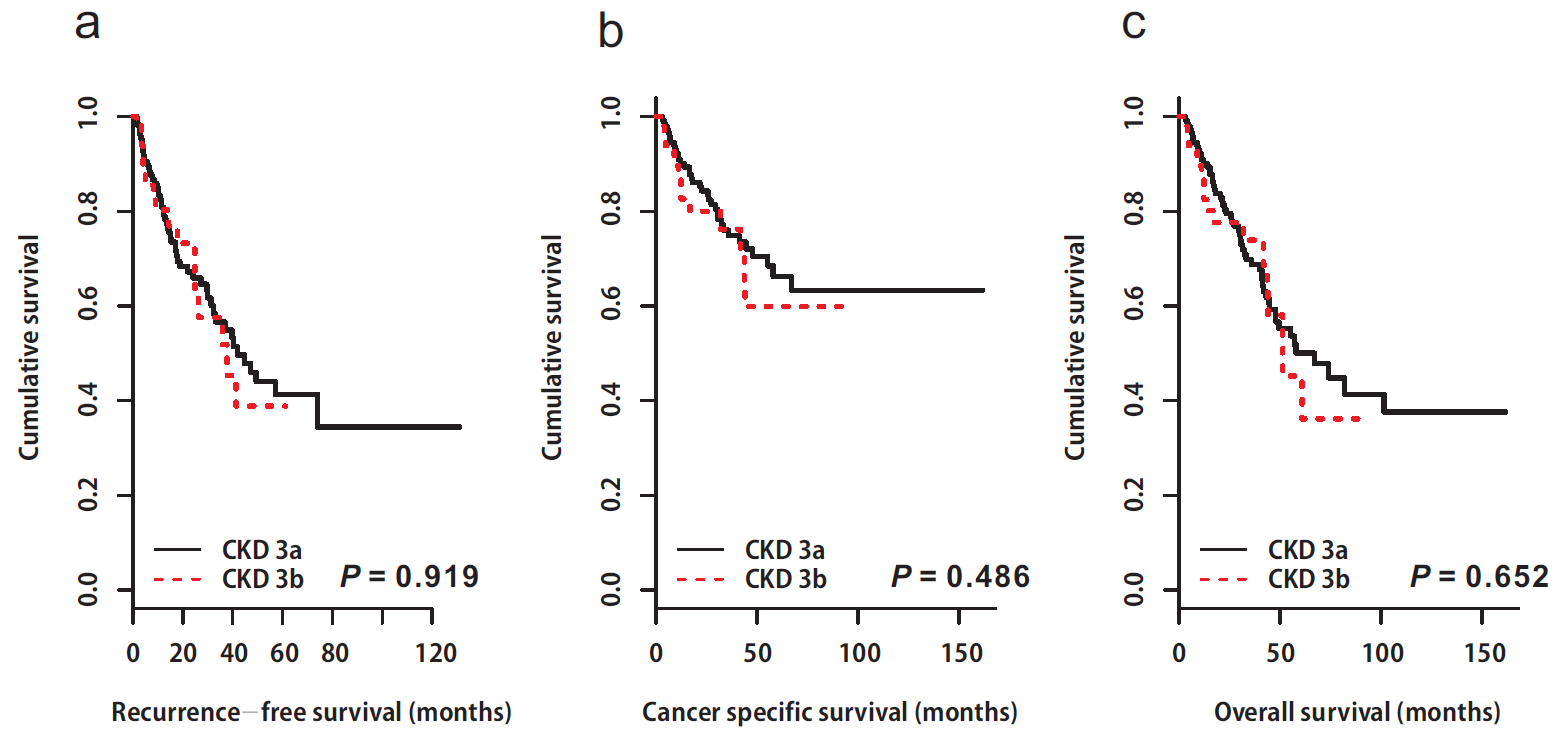
Kaplan-Meier estimates depicting the recurrence-free survival (a), cancer-specific survival (b), and overall survival (c) stratified according to chronic kidney disease stage 3 (3a vs. 3b) **a.** Recurrence-free survival. CKD 3a; median 82.3 (95% CI: not available) vs. CKD 3b; median: not available, *p* = 0.919. CKD, chronic kidney disease; CI, confidence interval. **b.** Cancer specific survival. CKD 3a; median: not available vs. CKD 3b; median: not available, *p* = 0.486. CKD, chronic kidney disease. **c.** Overall survival. CKD 3a; median: 67.0 months (95% CI: 47.9–not available) vs. CKD 3b; median: 51.3 months (95% CI: 43.7– not available), *p* = 0.652. CKD, chronic kidney disease; CI, confidence interval.

Following RNU, 71 (15.8%) patients died from UTUC. The 5-year CSS rates were 83.5% for the non-CKD group, and 65.1% for the CKD group (*p* = 0.001, Figure 1b). As with the results for RFS and CSS, the CKD group showed poor OS compared to the non-CKD group (5-year OS rates; non-CKD: 68.9% *vs*. CKD: 49.8%, *p* = 0.001, Figure 1c). Univariable and multivariable Cox regression analyses showed that preoperative CKD was associated with worse cancer-specific (univariable, HR: 2.76, 95% CI: 1.63-4.67, *p* = 0.001; multivariable, HR: 2.44, 95% CI: 1.44-4.14, *p* = 0.001; Tables 2 & 3) and overall survival (univariable, HR: 1.77, 95% CI: 1.23-2.56, *p* = 0.002; multivariable, HR: 1.66, 95% CI: 1.15-2.40, *p* = 0.007; Tables 2 & 3)In the subgroup analysis, there was no difference in survival between the two groups according to CKD stage 3 (3a *vs*. 3b, Figure 2).

## DISCUSSION

Our results showed that preoperative CKD is significantly associated with RFS, CSS, and OS in patients with UTUC. The independent predictive value of preoperative CKD persisted after multivariable Cox proportional hazard regression analysis. Patients with low preoperative eGFR ( < 60 mL/min/1.73m^2^) had worse RFS, CSS, and OS than those with higher eGFR (≥60 mL/min/1.73m^2^). Despite the fact that the exact mechanisms by which relative renal insufficiency leads to UTUC, and possibly to more aggressive UTUC, are unknown, there is some evidence from a population-based cohort study that demonstrates the association between decreased renal function and risk of urinary tract cancer including UTUC [12-14]. CKD is common in the elderly population and is suggested as an important risk factor for the development of many cancers including those of the prostate and urinary tract [12]. The incidence of urinary tract cancer in patients with moderate to severe CKD (stage III, IV, and V) was about three times greater than that in non-CKD patients. Hung et al. [13] showed a significant and proportional increase in the prevalence of UTUC with the severity of CKD. Among 267 patients with urothelial carcinoma, 11%, 55%, and 71% of those with none/mild CKD, CKD stage III, and CKD stage IV/V, respectively, had UTUC. Recently, a more large-scale and long-term follow-up study using a national health database in Taiwan demonstrated that CKD patients have an elevated risk for UTUC [14]. In the multivariate analysis, CKD patients had a 1.63-fold higher risk of UTUC than the non-CKD group (adjusted HR: 1.63, 95% CI: 1.26-2.13). However, the relationship between CKD and long-term oncologic outcomes such as RFS, CSS, and OS after RNU was not reported in the above studies.

The association between CKD and oncological outcomes was evaluated in several urinary tract cancers. A multicenter study from Korea demonstrated that preoperative CKD status was associated with lower RFS, CSS, and OS in renal cell carcinoma, and CKD was an independent predictor of CSS and OS in renal cell carcinoma [15]. Li et al. [16] reported that preoperative CKD was associated with worse RFS, progression-free survival, and OS in patients with non-muscle-invasive bladder cancer. CKD was also an independent risk factor for worse oncological outcomes in non-muscle-invasive bladder cancer patients. Tollefson et al. [17] evaluated the effect of preoperative CKD in prostate cancer patients who received radical prostatectomy. In their study, CKD affected deceased OS but not prostate cancer-specific survival. In addition, Chung et al. [18] showed that advanced CKD stage is associated with a greater risk of bladder recurrence following RNU. However, this was not a prognostic factor for CSS and OS. The authors explained that the discrepancy is due to the large percentage of low pathologic grade lesions in patients with CKD stage V. Furthermore, 69% of patients with CKD V had pT1 disease. In contrast, in our study, the percentage of patients with high pathologic grade lesions was higher in the CKD group than in the non-CKD group (77.2% *vs*. 63.8%).

Along with previous studies, our study clearly showed that CKD was related to poor oncological outcomes of UTUC such as RFS, CSS and OS. Interestingly, there was no difference in pathologic stage between the non-CKD and CKD groups; however, severe hydronephrosis, high-grade tumor, and lymphovascular invasion showed a stronger relationship with the CKD group. Among these variables, tumor stage and lymphovascular invasion were also independent prognostic factors for oncological outcomes in this study. Therefore, CKD may be related to aggressive UTUC [14].

As we have already mentioned, the precise biological explanation for the association between CKD and oncological outcomes in patients with UTUC is not well understood and may be multifactorial. One possible explanation is that chronic irritation which was caused by diminished urinary washing effect and accumulation of oncogenic metabolized nephrotoxic substances in the upper urinary tracts in CKD patients may contribute to a more aggressive pattern of UTUC [19]. In addition, CKD is also a proinflammatory condition [20], and there are several studies that have shown that markers of inflammation, such as C-reactive protein (CRP), have also been associated with oncological outcomes in patients with UTUC who underwent RNU [21]. Morizane et al. [21] reported that high preoperative CRP level and low preoperative eGFR ( < 50 mL/min/1.73m^2^) predict poor oncological outcomes in patients with UTUC. In this regard, it is likely that an association between CKD and inflammation results in poor oncological outcomes in UTUC. Unfortunately, in the present study, we did not evaluate markers of inflammation, such as CRP and white blood cell count. Another possible mechanism was suggested by Li et al. [22]. They suggested that the suppressed immunological setting in patients with UTUC with CKD may result in reduction of the capability of immune cells to recognize tumor-associated antigens, to undergo activation, and to bring into effect their antitumor activities, thereby fostering tumor progression. However, this hypothesis is based on immunologic defects in patients undergoing hemodialysis; therefore, it should be elucidated in CKD patients not undergoing hemodialysis.

Several concurrent complications are associated with decreased categories of GFR including infection, impaired cognitive and physical function, and threats to patient safety. Therefore, recently, CKD stage 3 was divided into CKD stage 3a (eGFR 59-45 ml/min/1.73m^2^) and 3b (eGFR 44-30 ml/min/1.73m^2^) [23]. It is believed that CKD 3b would be worse clinical outcome in general population. However, there was no survival difference between the CKD stage 3a and 3b patients in current study.

This study has several limitations. First, data were collected retrospectively at multiple centers. Variations among several surgeons and pathologists may exist. The pathologic reviews were not centralized. However, urologic pathologists determined all pathologic features at each institution. Lymph node invasion, which could affect the oncologic outcomes, was not examined. Second, we defined only decreased preoperative eGFR < 60 mL/min/1.73m^2^ calculated with the MDRD equation with serum creatinine as CKD. CKD is defined strictly as abnormalities of kidney structure or function, present for over 3 months, with implications for health. Criteria for CKD are abnormal kidney damage markers or decreased GFR [24]. The duration of CKD and other markers of kidney damage such as albuminuria and abnormal urine sediment should be considered to rule out acute kidney injury. In our study, we followed the Kidney Disease Outcomes Quality Initiative (KDIGO) guidelines, which mention that CKD can be ascertained by means of simple laboratory tests without identification of the cause of disease [24]. Third, preoperative risk factors for CKD were not investigated. CKD can be induced by many causes such as glomerulonephritis, tobacco use, herbal medicine, and UTUC itself. Only HTN and DM was investigated in the current study, and there was no difference between the non-CKD and CKD groups. Fourth, our death records were limited to information on whether the patient is dead or alive and if the death was cancer-related. Detailed information regarding competing causes of death was unavailable. Therefore, we could not assume that CKD leads to a high incidence of cardiovascular disease, resulting in early death. We tried to avoid the limitations of “respective analysis” such as uneven patient distribution among groups; thus, our analysis was conducted using 1:1 propensity matching. Well-designed prospective, randomized trials might be required to delineate the clear relationship between CKD and UTUC. Despite these limitations, our study population was relatively larger than those included in previous studies, and to our knowledge, this study represents the first attempt to determine whether preoperative CKD can predict oncologic outcomes in Korean patients with UTUC.

## MATERIALS AND METHODS

### Patient population

A total of 566 patients with ipsilateral UTUC who were treated using RNU with bladder cuff excision at the 6 tertiary medical centers from January 2004 to August 2014 were reviewed. Demographic data included age, sex (female *vs*. male), body mass index (BMI), diabetes mellitus (DM), hypertension (HTN), Eastern Cooperative Oncology Group performance status (ECOG-PS), tumor location (renal pelvis *vs*. ureter), tumor size, multifocality, operative method (laparoscopy *vs*. open), presence of synchronous bladder tumor, hydronephrosis, final pathological findings, adjuvant chemotherapy, and relapse pattern (if any). Patients diagnosed with previous bladder cancer, regional lymph node metastasis (solely on imaging study) or distant metastasis, and those treated with neoadjuvant chemotherapy were excluded from this study.

The estimated glomerular filtration rate (eGFR) was calculated by the Modification of Diet in Renal Disease (MDRD) Study equation (GFR in mL per minute per 1.73 m^2^ = 186 * sCr^-1.154^* age^-0.203^* (0.742 if female) * (1.210 if black)) [25]. Baseline eGFR was defined as stage I (≥ 90 mL/min/1.73m^2^), II (60-89 mL/min/1.73m^2^), III (30-59 mL/min/1.73m^2^), IV (15-29 mL/min/1.73m^2^), and V ( < 15mL/min/1.73m^2^) [26]. Then, the patients were assigned to non-CKD (Stage I and II, *n* = 342) and CKD (Stage III, IV, and V, *n* = 244) groups. This study was approved by the Institutional Review Boards of all the participating centers.

### Pathology

Tumor grade was determined using the 1998 World Health Organization (WHO) grading system and tumor staging was determined according to the 7th edition of the American Joint Committee on Cancer TNM classification by a variety of urological pathologists.

### Follow-up

All patients were evaluated postoperatively, *via* physical examination and radiologic imaging, every 3-4 months during the first year following RNU, every 6 months from the second through the fifth year, and annually thereafter. Recurrence was defined as local recurrence in the tumor bed, lymph node metastasis, or distant metastasis. Survival time was calculated from the date of radical surgery to final follow-up or death (cancer-specific or any other causes).

### Statistical analysis

Baseline patient demographics were analyzed using descriptive statistics. The Mann-Whitney U test and chi-square test were used for comparisons between groups of continuous and categorical variables, respectively. To reduce the effects of selection bias and potential confounding factors, such as baseline characteristics or uneven patient distribution between the non-CKD and CKD groups, we used 1:1 propensity score matching to adjust for age, sex, BMI, DM, and ECOG-PS [27]. Of the 342 patients in the non-CKD group, 224 were selected by propensity score matching analysis and compared with the 224 patients in the CKD group.

The effects of CKD on recurrence-free survival (RFS), cancer-specific survival (CSS), and overall survival (OS) were estimated using Kaplan-Meier survival plots with the log-rank test within the post-propensity-matched cohort. Factors associated with RFS, CSS, and OS were determined by univariable and multivariable Cox proportional hazard regression models. Among the factors, those with *p* < 0.25 were selected (on univariable analysis for RFS, CSS and OS) and included in the multivariable regression analysis (stepwise backward procedure) using Cox proportional hazards regression model, which was performed to achieve adjusted hazard ratio (HR) to determine independent prognostic factors for RFS, CSS and OS. All statistical analyses were performed using SPSS version 21.0 (IBM Corporation, Armonk, NY, USA), R version 2.14.2 software (R Foundation for Statistical Computing, Vienna, Austria, http://www.R-project.org), and MedCalc Statistical Software version 15.11.4 (MedCalc Software bvba, Ostend, Belgium; https://www.medcalc.org; 2015) with *p* values < 0.05 considered statistically significant.

## CONCLUSIONS

In this retrospective study including a large case series of patients who underwent RNU for UTUC, we observed that patients with preoperative CKD had worse recurrence-free survival, cancer-specific survival, and overall survival. Preoperative CKD is an important independent prognostic factor for oncologic outcomes in patients with UTUC who underwent RNU with bladder cuff excision. In patients with UTUC, CKD should be intensively managed before treatment, and CKD status should be considered in risk stratification for UTUC.

### Ethical standards

All procedures performed in studies involving human participants were in accordance with the ethical standards of the institutional research committee. The institutional ethics committee approved this study, and Informed consent was waived by the board.
